# Supplementary material for: Clinical phenotypes of chronic cough categorised by cluster analysis
Source: PLoS One. 2023 Mar 17;18(3):e0283352. doi: 10.1371/journal.pone.0283352 (PMC10022767; doi:10.1371/journal.pone.0283352)
Supplement: S1 Table — (DOCX) [file pone.0283352.s001.docx]

S1 Table. Correlation matrix of each aetiology with COAT (A) and K-LCQ (B) scores

(A)

|  | **UACS** | **Asthma/CVA** | **EB** | **GERD** |
| --- | --- | --- | --- | --- |
| Age | -0.062 | 0.044 | -0.023 | 0.093 |
| Female sex | -0.037 | 0.173 | 0.025 | -0.035 |
| COAT 1 | 0.034 | 0.112 | -0.093 | -0.096 |
| COAT 2 | 0.067 | 0.122 | -0.117 | -0.143 |
| COAT 3 | 0.052 | 0.232 | -0.067 | -0.212 |
| COAT 4 | -0.020 | 0.207 | -0.060 | -0.181 |
| COAT 5 | 0.033 | 0.101 | -0.109 | -0.181 |
| COAT total | 0.042 | 0.207 | -0.113 | -0.216 |

(B)

|  | **UACS** | **Asthma/CVA** | **EB** | **GERD** |
| --- | --- | --- | --- | --- |
| LCQ 1 | -0.089 | -0.063 | 0.118 | 0.031 |
| LCQ 2 | -0.095 | -0.160 | 0.121 | 0.187 |
| LCQ 3 | -0.004 | -0.198 | 0.081 | 0.144 |
| LCQ 4 | -0.050 | -0.127 | 0.093 | 0.080 |
| LCQ 5 | -0.039 | -0.141 | 0.089 | 0.175 |
| LCQ 6 | -0.014 | -0.120 | 0.027 | 0.135 |
| LCQ 7 | -0.039 | -0.175 | 0.129 | 0.170 |
| LCQ 8 | -0.057 | -0.165 | 0.139 | 0.169 |
| LCQ 9 | -0.035 | -0.177 | 0.099 | 0.149 |
| LCQ 10 | -0.017 | -0.160 | 0.021 | 0.142 |
| LCQ 11 | -0.066 | -0.078 | 0.111 | 0.107 |
| LCQ 12 | -0.041 | -0.107 | 0.071 | 0.138 |
| LCQ 13 | -0.060 | -0.089 | 0.066 | 0.206 |
| LCQ 14 | -0.189 | -0.115 | 0.204 | 0.182 |
| LCQ 15 | 0.017 | -0.105 | 0.081 | 0.064 |
| LCQ 16 | -0.069 | -0.064 | 0.120 | 0.069 |
| LCQ 17 | -0.067 | -0.086 | 0.088 | 0.177 |
| LCQ 18 | -0.040 | -0.018 | 0.026 | 0.077 |
| LCQ 19 | -0.061 | -0.052 | 0.075 | 0.174 |
| LCQ total | -0.077 | -0.163 | 0.136 | 0.200 |

UACS, upper airway cough syndrome; CVA, cough variant asthma; EB, eosinophilic bronchitis; GERD, gastroesophageal reflux disease
