# Supplementary material for: Clinical phenotypes of chronic cough categorised by cluster analysis
Source: PLoS One. 2023 Mar 17;18(3):e0283352. doi: 10.1371/journal.pone.0283352 (PMC10022767; doi:10.1371/journal.pone.0283352)
Supplement: S2 Table — (DOCX) [file pone.0283352.s002.docx]

S2 Table. Detailed K-LCQ scores among clusters in patients with chronic cough

|  | **Total** | **Cluster 1** | **Cluster 2** | **Cluster 3** | **Cluster 4** |
| --- | --- | --- | --- | --- | --- |
| **LCQ total** | 11.2 ± 3.1 | 14.5 ± 2.8* | 12.7 ± 2.3* | 9.2± 1.9* | 9.7 ± 2.7* |
| LCQ1 | 4.7 ± 1.4 | 5.4 ± 1.5* | 4.8 ± 1.3 | 4.3 ± 1.4* | 4.8 ± 1.4 |
| LCQ2 | 3.6 ± 1.7 | 4.6 ± 1.7* | 3.9 ± 1.5 | 3.0 ± 1.6* | 3.3 ± 1.7 |
| LCQ3 | 3.9 ± 1.5 | 5.2 ± 1.3* | 4.9 ± 1.1* | 2.9 ± 1.0* | 3.3 ± 1.4* |
| LCQ4 | 2.7 ± 1.3 | 3.8 ± 1.7* | 3.3 ± 1.2* | 1.9 ± 0.7* | 2.2 ± 0.9* |
| LCQ5 | 3.6 ± 1.7 | 5.0 ± 1.1* | 4.2 ± 1.1* | 2.8 ± 0.9* | 3.1 ± 1.2* |
| LCQ6 | 3.1 ± 1.4 | 4.2 ± 1.4* | 3.7 ± 1.3* | 2.4 ± 1.0* | 2.7 ± 1.3* |
| LCQ7 | 3.7 ± 1.5 | 5.2 ± 1.1* | 4.3 ± 1.2* | 2.8 ± 1.0* | 3.2 ± 1.4* |
| LCQ8 | 3.6 ± 1.4 | 4.9 ± 1.1* | 4.3 ± 1.1* | 2.8 ± 1.1* | 3.1 ± 1.4* |
| LCQ9 | 4.1 ± 1.6 | 4.6 ± 1.5* | 4.6 ± 1.4* | 3.9 ± 1.5 | 3.4 ± 1.2* |
| LCQ10 | 4.2 ± 1.5 | 4.8 ± 1.5* | 5.1 ± 1.1* | 3.8 ± 1.6* | 3.6 ± 1.3* |
| LCQ11 | 3.8 ± 1.3 | 4.6 ± 1.4* | 4.3 ± 1.2* | 3.2 ± 1.0* | 3.4 ± 1.2* |
| LCQ12 | 4.4 ± 1.5 | 5.2 ± 1.4* | 5.0 ± 1.3* | 4.0 ± 1.4* | 3.8 ± 1.5* |
| LCQ13 | 3.3 ± 1.6 | 4.8 ± 1.4* | 3.9 ± 1.5* | 2.4 ± 1.1* | 2.9 ± 1.4* |
| LCQ14 | 4.9 ± 1.4 | 5.7 ± 1.1* | 5.4 ± 1.1* | 4.5 ± 1.4* | 4.3 ± 1.6* |
| LCQ15 | 3.3 ± 1.5 | 4.1 ± 1.7* | 3.6 ± 1.5 | 2.9 ± 1.2* | 2.9 ± 1.5* |
| LCQ16 | 3.5 ± 1.6 | 4.3 ± 1.4* | 3.9 ± 1.5 | 3.2 ± 1.5* | 3.1 ± 1.6* |
| LCQ17 | 3.6 ± 1.6 | 4.8 ± 1.3* | 3.8 ± 1.6 | 2.4 ± 1.3* | 3.3 ± 1.5 |
| LCQ18 | 4.0 ± 1.6 | 5.1 ± 1.3* | 4.5 ± 1.4* | 3.3 ± 1.4* | 3.5 ± 1.5* |
| LCQ19 | 3.2 ± 1.7 | 4.8 ± 1.3* | 3.6 ± 1.6* | 2.4 ± 1.3* | 2.7 ± 1.4* |

Physical domain section includes questions about chest/stomach pain, accompany of bothersome phlegm, tiredness, hypersensitivity to irritants, sleep difficulties, frequency of coughing bouts, presence of voice hoarseness, and loss of energy due to cough. In psychological domain, questions of feeling fed-up, worrying about serious illness, and concerns of what other people might think are included. Social domain contains questions of interference with job or daily tasks, life enjoyment, interruption of telephone call conversation, and annoyance of partner, family, or friend.
