# Supplementary figures and images for: Clinical phenotypes of chronic cough categorised by cluster analysis
Source: PLoS One. 2023 Mar 17;18(3):e0283352. doi: 10.1371/journal.pone.0283352 (PMC10022767; doi:10.1371/journal.pone.0283352)

S1 Fig. Histogram of COAT questionnaire


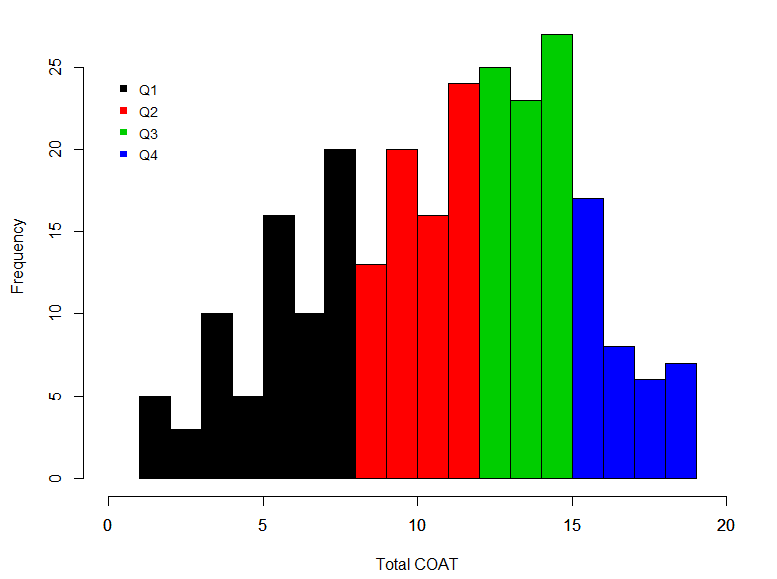


Q1: 1.0-8.0, Q2: 8.0-12.0, Q3: 12.0-15.0, Q4: 15.0-19.0

Supplement: S1 Fig — (DOCX) [file pone.0283352.s003.docx]
