# Supplementary material for: Clinical phenotypes of chronic cough categorised by cluster analysis
Source: PLoS One. 2023 Mar 17;18(3):e0283352. doi: 10.1371/journal.pone.0283352 (PMC10022767; doi:10.1371/journal.pone.0283352)

S2 Fig. Prevalence of causes of chronic cough (A) and Venn Diagram (B)

(A)


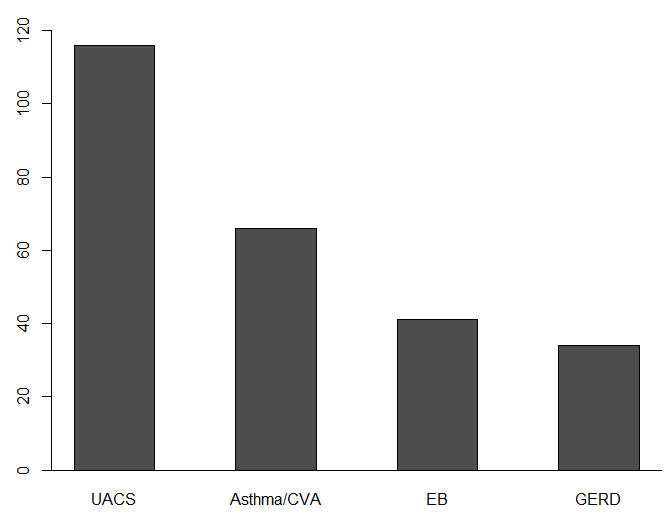


UACS, upper airway cough syndrome; CVA, cough variant asthma; EB, eosinophilic bronchitis; GERD, gastroesophageal reflux disease

(B)


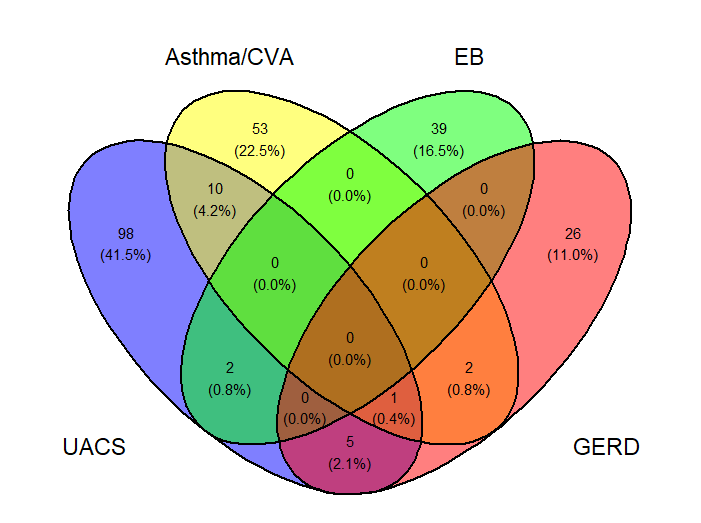

Supplement: S2 Fig — (DOCX) [file pone.0283352.s004.docx]
