# Supplementary material for: Clinical phenotypes of chronic cough categorised by cluster analysis
Source: PLoS One. 2023 Mar 17;18(3):e0283352. doi: 10.1371/journal.pone.0283352 (PMC10022767; doi:10.1371/journal.pone.0283352)
Supplement: S3 Fig — (DOCX) [file pone.0283352.s005.docx]

S3 Fig. Plots of silhouette width according to cluster number

**
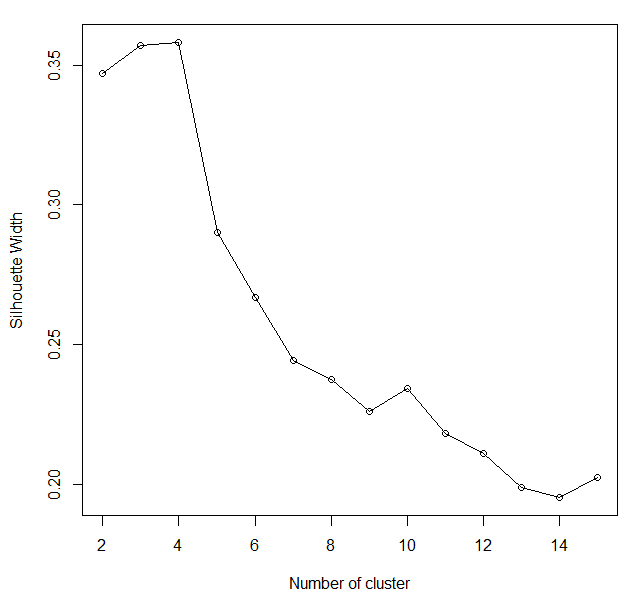
**

Distance for silhouette width was measured using the cluster package of R software (version 3.6.0)
