# Supplementary material for: Clinical phenotypes of chronic cough categorised by cluster analysis
Source: PLoS One. 2023 Mar 17;18(3):e0283352. doi: 10.1371/journal.pone.0283352 (PMC10022767; doi:10.1371/journal.pone.0283352)
Supplement: S4 Fig — (DOCX) [file pone.0283352.s006.docx]

S4 Fig. Distribution of each cluster and their correlations between age and COAT score


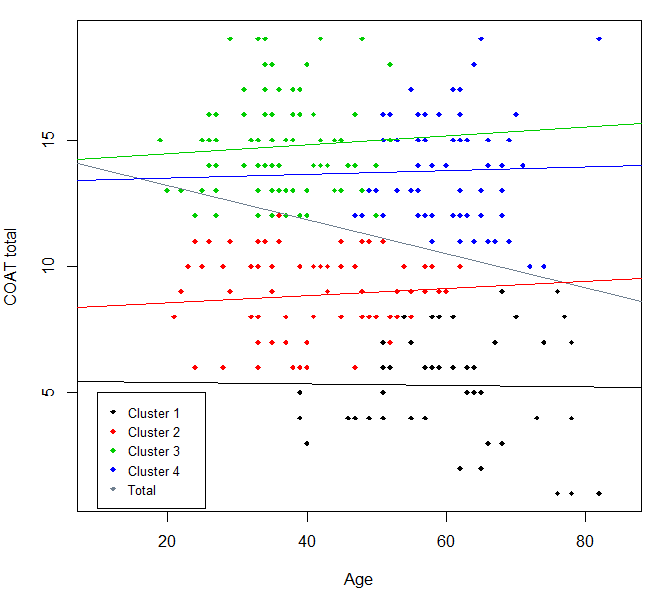


Correlation between age and total COAT score in cluster 1 (P=0.459), cluster 2 (P=0.925), cluster 3 (P=0.557), and cluster 4 (P=0.838) were not significant.

Different colour indicates each cluster (black: cluster 1, red: cluster 2, green: cluster 3, and blue: cluster 4)
