# Supplementary material for: Clinical phenotypes of chronic cough categorised by cluster analysis
Source: PLoS One. 2023 Mar 17;18(3):e0283352. doi: 10.1371/journal.pone.0283352 (PMC10022767; doi:10.1371/journal.pone.0283352)
Supplement: Attachment — Submitted filename: Clinical Phenotypes of Chronic Cough Categorised by Cluster Analysis.docx [file pone.0283352.s008.docx]

Clinical Phenotypes of Chronic Cough Categorised by Cluster Analysis

1) elderly with mild 50  cough,

2) middle-aged with less severe cough, - no number

3) relatively male-predominant youth with 51  severe cough, and

4) female-predominant elderly with severe cough. – No numbers

Cluster 1 consisted of the oldest population but showed the least severe cough. In this 224  cluster, GERD contributed to a higher proportion of the underlying causes of cough than in 225  the others.

Cluster 2 was composed of a wide range of ages with medium cough severity 226  and a middle-aged mean age value. This cluster had the lowest proportion of asthma/CVA. 227  Although both clusters

3 and 4 consisted of patients with the most severe cough, different

228  patterns in demographics and underlying diseases were observed.

No predominance phenotype of younger women?

It would have a better clinical usage if we could link the score/phenotype to possible causal etiology

Females seem to predominate all categories in Table1 contrary to text?

COAT is said to be simpler, Did you attempt to see if categorization would using the Korean version?

There is need for an expert in analytics to review the paper otherwise a very good paper that is making first step to move us to the future
