# Supplementary material for: Clinical phenotypes of chronic cough categorised by cluster analysis
Source: PLoS One. 2023 Mar 17;18(3):e0283352. doi: 10.1371/journal.pone.0283352 (PMC10022767; doi:10.1371/journal.pone.0283352)
Supplement: Attachment — Submitted filename: Response to reviewer_R2.docx [file pone.0283352.s010.docx]

**ACADEMIC EDITOR:**
Please proofread your paper thoroughly and correct spelling, punctuation, grammar, and formatting errors. Because all references will be linked electronically as much as possible to the papers they cite, proper formatting of references is crucial. For more information, please refer to submission guidelines of the journal.

Answer: Thank you very much for allowing us the opportunity to correct our errors. We corrected spelling, punctuation, grammar, and reference formatting errors all through the manuscript. Both tracked and clean versions of the manuscript are provided. Again, we appreciate all the invaluable comments that improved the overall quality of our manuscript.
